# OP16 Health Technology Assessment Framework In India: Challenges And Opportunities For Policy Enhancement

**DOI:** 10.1017/S0266462325100743

**Published:** 2025-12-29

**Authors:** Anupama Sharma, Ranjan Kumar Choudhury, Yasmin Khan

## Abstract

**Introduction:**

Health technology assessment (HTA) evaluates the cost effectiveness and clinical efficacy of health technologies to inform policy decisions. Established in 2017 by the Department of Health Research, the Office of Health Technology Assessment in India is housed within the Ministry of Health and Family Welfare; however, challenges remain. This study analyzed gaps in India’s HTA framework to aid policymakers in making improvements.

**Methods:**

The study conducted a systematic review of HTA literature and a focus group discussion with Ministry of Health representatives. A search on PubMed using “HTA” AND “India” yielded 74 papers from the last seven years, with 12 additional papers found through Google. Using the PRISMA methodology, titles, abstracts, and full texts were evaluated, and duplicates were managed with Zotero. Data were collected in an excel sheet, and the HTA scorecard from Türkiye helped identify context-specific gaps in India, which were discussed with six focus-group members from the HTA department at the Ministry of Health and Family Welfare and the National Health Systems Resource Centre.

**Results:**

The desk review identified gaps in India’s data usage, including a fragmented data ecosystem, limited electronic health records, and challenges with real-world evidence that affect HTA effectiveness. A major issue was the scarcity of trained professionals for high quality HTA and insufficient public funding for HTA research. The potential of HTA remains underutilized. Barriers to effective HTA legislation include the complexity of central policies, incoherence with state administration, and fragmented insurance schemes. Focus group participants recommended capacity building and orientation programs for the HTA department and state procurement, highlighting the need for funding to support implementation research for benchmark development.

**Conclusions:**

India faces challenges in data infrastructure, stakeholder involvement, funding, and policy integration for HTA. Investments in data collection and training are essential. With initiatives such as Digital India and Make in India, the country aims to establish itself as a global digital economy. Strengthening HTA institutionalization and implementing an artificial intelligence-based HTA framework can position India as a leader in HTA.

